# Wiggle and glide: fine-scale telemetry reveals unique diving strategies in benthic-foraging sea snakes

**DOI:** 10.1186/s40462-025-00592-z

**Published:** 2025-08-28

**Authors:** Shannon Coppersmith, Claire Goiran, Kate Laura Sanders, Jenna Margaret Crowe-Riddell, Olivier Chateau, Richard Shine, Vinay Udyawer

**Affiliations:** 1 School of Biological Sciences, The University of Adelaide, Adelaide, South Australia Australia; 2 ISEA & LabEx Corail, Université de La Nouvelle-Calédonie, Nouméa, New Caledonia New Caledonia; 3 Laboratory of Marine Biology and Ecology, Aquarium des Lagons, Nouméa, New Caledonia New Caledonia; 4 School of Natural Sciences, Macquarie University, Sydney, NSW Australia; 5 Sharks Pacific, Rarotonga, Cook Islands

**Keywords:** Biologging, Energetics, Behaviour, Sensory, Acoustic telemetry

## Abstract

**Background:**

The efficient acquisition of two critical but spatially separated resources –food and oxygen– governs the daily movements and diving patterns of air-breathing aquatic animals. Unlike pinnipeds, turtles and seabirds, fully marine (‘true’) sea snakes spend their entire lifecycle at sea and have evolved specialised movement behaviours. However, fine-scale data on the diving behaviour of free-ranging sea snakes remain scarce, limiting our understanding of their ecology and vulnerability to anthropogenic threats.

**Methods:**

We used acoustic telemetry to track five individuals of two benthic-foraging sea snake species (*Hydrophis stokesii*,* H. major*) in Exmouth Gulf, Western Australia, and Baie des Citrons, New Caledonia. Each snake was continuously tracked using a directional hydrophone for up to 18 h, generating high-resolution, three-dimensional dive paths. After filtering, we analysed 106 dives from 46 h of tracking.

**Results:**

Sea snakes primarily conducted U- and S-shaped dives and spent on average 97.2% of their time submerged. Most U-shaped dives were characterised by limited vertical and horizontal movement. S-shaped dives were more complex, with variable time on the seafloor and occasionally interrupted gradual ascents. Dive duration was positively correlated with post-dive surface interval, while depth and duration of the gradual ascent phase were influenced by environmental depth. We also identified distinctive, repetitive undulations (‘wiggles’) in the depth profiles of several dives completed by all three tracked *H. stokesii*.

**Conclusions:**

These high-resolution data provide the first insights into the fine-scale diving patterns of benthic-foraging sea snakes. Like surface-foraging species, they appear to regulate air intake based on environmental depth and may be neutrally buoyant in the gradual ascent phase of S-shaped dives. We hypothesise that this phase facilitates efficient horizontal travel, despite potential increases in predation risk. The ‘wiggles’ observed in *H. stokesii* may have a functional role in buoyancy control, energy conservation, or foraging. Our study contributes to a deeper understanding of sea snake diving strategies, with implications for their ecology, physiology, and conservation.

**Supplementary Information:**

The online version contains supplementary material available at 10.1186/s40462-025-00592-z.

## Background

The movement of individual organisms is a vital component of life, closely associated with nearly all ecological and evolutionary processes. It impacts the acquisition of resources and shapes the structure and dynamics of populations, communities and ecosystems [1]. Consequently, patterns in animal movement are inherently complex, driven by processes that act across a range of spatial and temporal scales. For instance, on a diel timescale, animal movements are primarily governed by the need to find food, mates, suitable habitat and to avoid predators [2]. For secondarily aquatic animals, movement is further complicated by the spatial separation of food and oxygen in their three-dimensional environment [3–5]. Investigating the daily movements to access these resources through the study of diving behaviour can clarify broader movement patterns, such as dispersal and migration, in air-breathing aquatic species.

All breath-hold diving animals must balance their energetic budgets with the replenishment of oxygen stores at the water’s surface [6]. However, this is especially crucial for so-called ‘surfacers’. Unlike ‘divers’ who spend extended periods on land and dive only to feed (e.g. seabirds and marine iguanas), surfacers remain submerged for many other key behaviours such as courtship and mating, travelling, and resting, and often remain at sea for their entire life cycle (e.g. cetaceans and sea snakes) [5, 7]. Their success is therefore dependent on their ability to maximise the benefit gained from underwater activities whilst minimising the energetic cost [5]. Correspondingly, surfacers often perform multiple tasks (e.g. resting and foraging) in a single dive [8] and may even alter their original objective partway through a dive based on their success [9, 10]. Fine-scale movement data are needed for distinguishing such complex and variable behaviours [4]. Although bio-logging has been used extensively to collect these movement data in a range of surfacers, including pinnipeds, turtles and cetaceans, the behaviours of many small and/or elusive species remain largely unknown [11 and references therein].

One such group are the fully aquatic ‘true’ sea snakes in the viviparous, front-fanged Hydrophiinae. All but three mangrove dwelling species of the > 60 species of true sea snakes are surfacers, never leaving the ocean and spending most of their time underwater [12]. They possess a range of specialised adaptations for aquatic life, including dorsoventrally elongated bodies and paddle-like tails for anguilliform swimming [13], salt-excreting glands, and sealable (haemostatic) nostrils [14]. Sea snakes have prolonged voluntary dive durations of up to 3.6 h facilitated by increased lung volume, cutaneous gas exchange, breathing tachycardia, and cardiac shunting [15]. Cutaneous respiration plays a significant role in extending dive times, with up to 30% of total oxygen uptake and most CO₂ elimination occurring across the skin in some species [12]. This capacity is enhanced by highly vascularised skin and a flattened body shape that increases surface area relative to volume [13]. Sea snakes form an important component of many tropical and sub-tropical marine ecosystems, serving as both prey for top predators such as tiger sharks, large predatory fishes, and seabirds, and as predators themselves with dietary specialisations ranging from fish eggs to large moray eels [12]. Despite their ecological significance, research on the fine-scale behavioural ecology of sea snakes has remained limited, largely due to the challenges of locating, capturing, and handling these elusive and venomous marine reptiles.

While a growing body of research has focused on the long-term and broad-scale movements of sea snakes [e.g., 16, 17, 18]; fine-scale data at a resolution sufficient to examine diving behaviour has only been collected and analysed for the yellow-bellied sea snake (*Hydrophis platurus*) [15]. This pioneering study was later reanalysed by Cook & Brischoux [19], who found that *H. platurus* spent 95% of their time underwater, diving to depths of up to 50 m and remaining submerged for as long as 3.6 h. Most dives were S-shaped (i.e. sinusoidal in shape when viewed in a time-depth profile), a dive shape that has since only been observed in sea turtles [e.g., 8, 20–22]. However, *H. platurus* is the only pelagic and ‘planktonic’ species of sea snake, feeding exclusively at the surface, and is therefore unlikely to be representative of species that forage along the seafloor. All other true sea snakes are benthic-foragers restricted to coastal areas, and their diving behaviour remains poorly understood, with existing knowledge limited to a few anecdotal or observational reports made from boats, snorkellers, or divers [9].

Improved understanding of benthic-foraging sea snakes’ dive behaviour is essential to assess how they access resources, avoid predators, and manage energetic demands in dynamic coastal environments. Fine-scale dive data can also offer valuable insights into how these species may respond to environmental change, as warming oceans and shifting prey distributions could alter the energetic costs and benefits of different underwater behaviours [23]. The present study was therefore motivated by a need to better understand how benthic-foraging sea snakes use their underwater space at fine scales. Specifically, we aimed to: (1) characterise the dive profiles of benthic-foraging true sea snakes, (2) investigate the function of key dive phases, and (3) characterise any novel diving behaviours identified in fine-scale dive profiles.

## Methods

### Study species and location

**Fig. 1:**
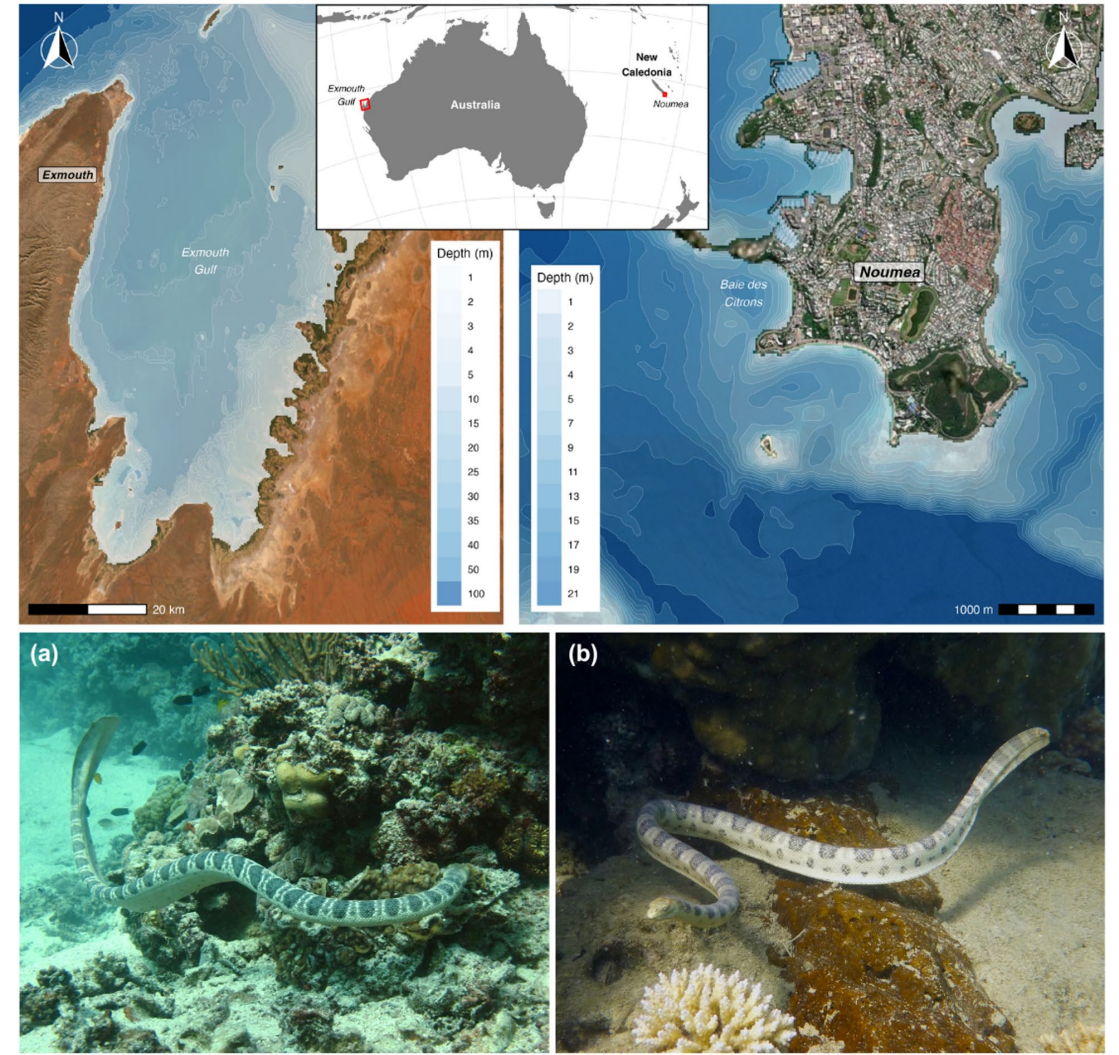
Maps of study locations Exmouth Gulf, Western Australia and Baie des Citrons, New Caledonia (top panels) with shades of blue indicating depth, overlaid on Esri World Imagery basemaps [34], and study species (**a**) *Hydrophis stokesii* and (**b**) *Hydrophis major* (bottom panels). Photographs by (**a**) Australian Institute of Marine Science, and (**b**) Claire Goiran

Sea snakes were manually tracked using acoustic telemetry in two locations: the west coast of Exmouth Gulf, Western Australia and Baie des Citrons in New Caledonia (Fig. 1). Exmouth Gulf is a large (2600 km^2^) embayment in the north-west of Western Australia. Its shallow waters (< 23 m) are dominated by macroalgal beds, coral reefs, and sandy substrate [24, 25]. Much of the gulf consists of soft sediment habitats interspersed with shallow rocky and coral reef shoals, with a semi-contiguous fringing reef system along the north-east coast of the gulf. The eastern and central regions of the gulf also support a major bottom-trawling prawn fishery in which sea snakes are regular by-catch [26]. Baie des Citrons is a small (~ 12.5 km^2^) shallow bay located beside the city of Noumea, in the archipelago of New Caledonia. The habitat within the bay comprises dense fringing reef corals and sandy inter-reefal patches, extending into deeper waters where the coral reefs transition to rocky substrates [27]. The habitat outside the bay extends to patchy coral and rocky reefs interspersed across a sandy substrate of up to 25–30 m in depth.

The two tagged species included in our analyses are fully aquatic benthic foragers. Stokes’ sea snake (*Hydrophis stokesii*) is a heavy-bodied generalist that primarily feeds on benthic fish such as toadfishes, many of which are toxic [28, 29]. One of the bulkiest species of sea snake, it can reach up to 2 m in length and 28 cm in girth [12]. *H. stokesii* is widely distributed, occurring in the coastal waters of the Indo-Pacific, between the Arabian Gulf and Northern Australia [30]. Although still a large-bodied species, the greater sea snake, also known as the olive-headed sea snake (*Hydrophis major*, previously *Disteira major*), grows to just 1.5 m in length and is more slender than *H. stokesii* [31]. They are believed to feed exclusively on striped eel catfish (*Plotosus lineatus*), which swim in dense swarms as juveniles but are more solitary as adults [17, 32]. *Hydrophis major* is found in the coastal waters of Northern Australia and Southern Papua New Guinea between Shark Bay in Western Australia and New Caledonia [33].

### Data collection

Tracking was conducted in October 2017 in New Caledonia and between April 2022 and October 2023 in Exmouth Gulf. Sea snakes belonging to the species *Hydrophis stokesii* and *Hydrophis major* were captured either by hand or with a dip-net. Once captured, sex was determined by examining external morphological features (presence or absence of hemipenal bulges), and snout-vent length (SVL) and mass were recorded. Each snake was either implanted with a passive integrated transponder (PIT) or photographed for future identification.

Continuous acoustic transmitters (V9P-2x, Innovasea Ltd.; 9 mm diameter, 31 mm length, 2.8 g in water and neutrally buoyant) were surgically implanted into *H. major* in New Caledonia and externally attached to *H. stokesii* individuals in Exmouth Gulf (Table 1). Only individuals in good condition and exceeding a minimum body mass (280 g; tags less than 1% body weight) were used. External transmitters were attached dorsally, approximately halfway between snout and tail, using three subdermal nylon sutures (see Supplementary Information Fig. S1), whereas internal transmitters were implanted into the peritoneal cavity under 2-5 ml/kg of local anaesthetic (ilium^®^; 20 mg/ml lignocaine hydrochloride) following methods from Udyawer et al. [18]. Surgical implantation was used for *H. major* in New Caledonia as it followed previously approved methods for tracking sea snakes in that region. In contrast, external attachment was used for *H. stokesii* in Exmouth Gulf under a separate animal ethics approval, where more recent work (Udyawer et al., unpublished data) had demonstrated the success of this less invasive method.

If weather was poor or crew availability was limited at the time of capture, snakes were held in aquaria prior to tag attachment and release to maximise tracking time (see Supplementary Information Table S1 for summary of snake capture and release metadata). After tagging, snakes were released at their capture locations, except for one *H. stokesii* individual, which was released ~ 8.5 km due to poor conditions (the release site had similar habitat depth and seafloor characteristics but reduced swell and surface currents).

Each transmitter emitted pulses with a unique frequency pulse (between 60 and 81 kHz) every two seconds and was equipped with a pressure sensor that recorded and transmitted depth with each pulse. Pressure sensors used in New Caledonia had an accuracy of ± 1.7 m and a resolution of 0.2 m, whereas those deployed in Exmouth Gulf had higher accuracy (± 0.5 m) and finer resolution (0.15 m). Transmitter signals were monitored using a boat-mounted directional hydrophone (VH110; estimated range of 250–500 m in clear water) and receiver unit (VR-100) which recorded GPS positions and depth data. The hydrophone was positioned below the hull of the research vessel on a two-metre rotating pole, allowing for 360˚ directional tracking.

Tagged snakes were followed by homing in on the strongest signal, and efforts were made to maintain signal strengths above 80 decibels to ensure location accuracy. All research vessels were small (7–8 m long), allowing for precise tracking and minimal disturbance compared to larger vessels. GPS coordinates of the vessel, along with water temperature, depth, tidal height, and flow direction (i.e. rising or falling) were recorded every 15 min using the vessel’s navigation system. Snakes were tracked for as long as possible, though sessions were limited by crew availability, weather conditions and ability to remain in range of the tagged individual.

For snakes with external tags, attempts were made to recapture snakes at the end of the tracking period to remove transmitters. External attachments were designed with a corrodible aluminium link that detaches within ~ 2 weeks if recapture was unsuccessful. Internally implanted transmitters remain in the individual indefinitely.

### Dive analysis

All data analyses were conducted in the R Statistical Environment (v4.1.2) [35]. Data were pre-processed by filtering detections based on signal strength and depth to remove false detections. We excluded all detections greater than 0.5 m above sea level, as well as those exceeding the maximum depth recorded on the vessel sounder by more than 1 m for a given track. Signal strength thresholds varied between individuals due to differences in transmitter resolution and the influence of sea conditions on detection accuracy. Lower limits were chosen by testing a range of thresholds and selecting the minimum value that retained all surfacing events and dive shapes while excluding erroneous detections. The minimum signal strength retained in individual tracks ranged from 43 to 65 dB. A summary of the individual filtering thresholds is provided in Supplementary Table S2. We then applied a median smoothing filter to remove any detections that deviated by more than 2 m from the median depth within a moving window of ten consecutive detections. This process systematically removed remaining outliers. Overall, between 2.3 and 23.9% of detections were filtered from the raw data for each individual (Table S2). Finally, any dives recorded within the first 20 min of tracking were excluded to eliminate potential behavioural artefacts associated with post-release acclimation to the acoustic transmitter. Across individuals, this period consistently encompassed the time during which dive durations and profiles appeared irregular or inconsistent with subsequent behaviour. After this initial period, dive shapes, durations, and surfacing intervals stabilised, suggesting the snakes had resumed typical movement patterns.

Initial analyses of sea snake diving behaviour were performed using the package ‘diveMove’ [36], which is used to visualise depth data from time-depth recorders, zero-offset correct depth, and divide the record into different phases (descent, bottom, and ascent). A depth threshold of 0.5 m was used to identify dive events and zero offset corrections (ZOC) were applied to the depth record based on the depth when the snake was at the surface.

**Fig. 2:**
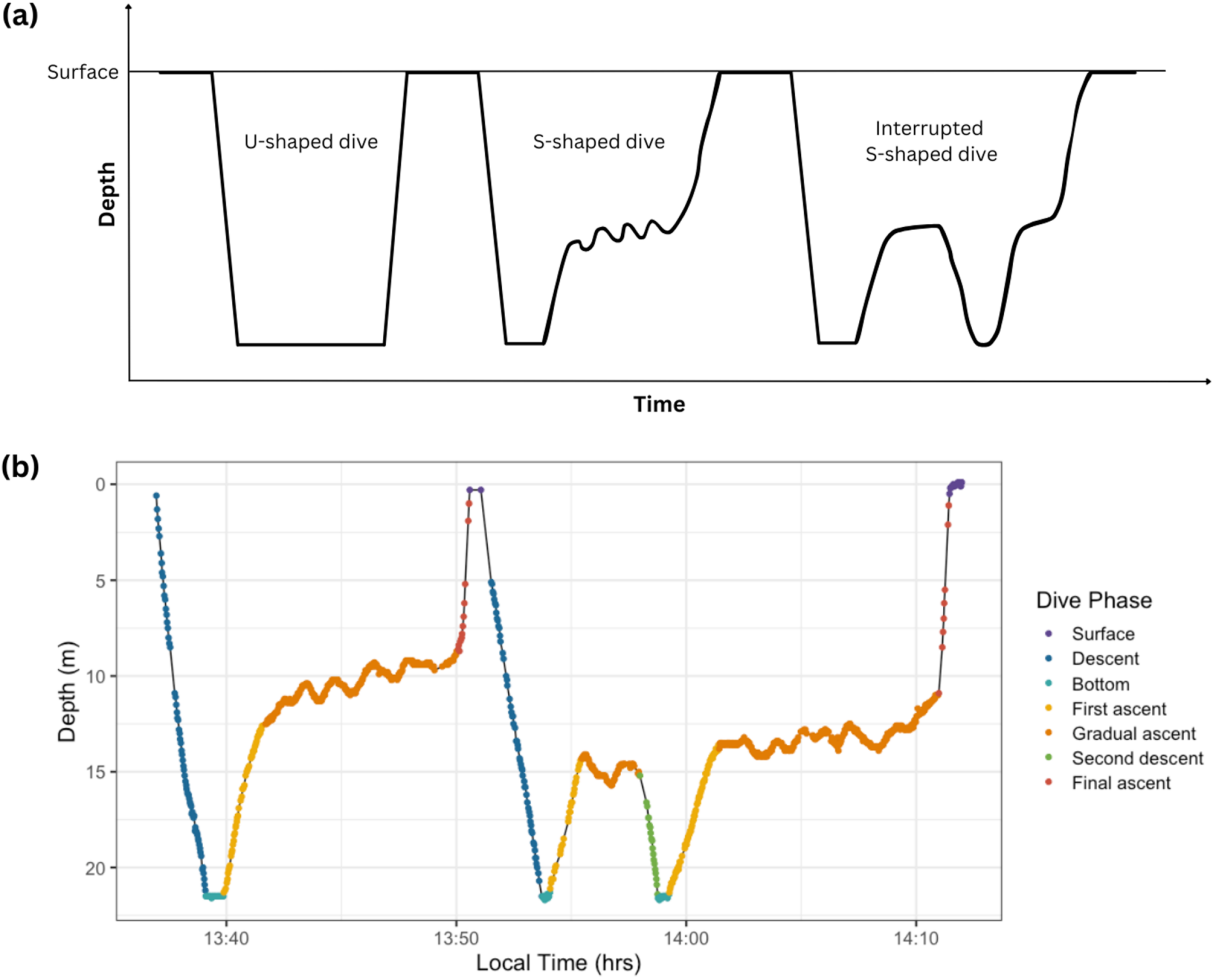
(**a**) Schematic of different dive types (U, S, and interrupted S). The repeated oscillation in the gradual ascent phase of the S-shaped dive represents 'wiggling' observed in several *H. stokesii* dives. (**b**) Time-depth profiles of an S-dive followed by an interrupted S-dive completed by *H. stokesii* ‘Cruise’ (also exhibiting wiggles), with colours representing each phase of the dive

Dives were visually categorised according to their dive shape in diveMove. Each dive was categorised as either U-shaped or S-shaped (Fig. 2a), categories previously used in studies on other air-breathing reptiles [8, 19, 37–39]. U-shaped (or flat-bottomed) dives are characterised by a rapid descent to maximum dive depth followed by a period of extended bottom time and concluded by a rapid ascent to the surface. S-shaped dives consisted of four or more phases and were defined by a characteristic ‘gradual ascent’ phase that has been described in previous literature on marine turtles and pelagic sea snakes [e.g., 8, 19–22]. The minimum four phases that make up an S-shaped dive include (1) a rapid ‘descent’ to the maximum dive depth, (2) a rapid ‘first ascent’ to the water column, (3) a ‘gradual ascent’ consisting of a slow, long ascent in the water column, and (4) a final quick ascent to the surface. S-shaped dives also sometimes included a period of bottom time following the initial descent, before the first ascent. We also identified more complex S-shaped dives, where the snake appeared to interrupt the gradual ascent phase by descending back to the bottom before returning to the same depth in the water column again. Hence, ‘interrupted S-shaped dives’ also included one or more secondary descents and ascents (Fig. 2b) but were grouped with other S-shaped dives in the analyses.

Dives and their phases (descent, bottom, ascent and post-dive surface interval) were initially identified using the *createTDR* and *calibrateDepth* functions in the ‘diveMove’ R package. However, the gradual ascent, first ascent and final ascent phases were not distinguishable in ‘diveMove’. We therefore manually classified these phases in all S- and interrupted S-shaped dives. Using these classifications, we calculated summary statistics relating to the depth and duration of each dive and phase. Dive duration was defined as the period of submergence between surfacing events, and post-dive surface interval as the surface period immediately following the dive. Descent rate was quantified as the descent distance (in metres) divided by the descent time (in seconds).

To assess fine-scale activity during the bottom phase of dives, we also calculated the standard deviation (SD) of depth for each individual U-shaped dive and identified any outliers using the 1.5 × interquartile range (IQR) rule. These per-dive SD values were then summarised by species to compare the variability in bottom-phase depth, which may reflect differences in activity such as resting versus foraging [40, 41].

Finally, we counted the number of undulations in the gradual ascent and bottom phases of individual dive profiles. In accordance with the literature on similar movement patterns in other air-breathing vertebrates, we defined a ‘wiggle’ as any event involving a rapid oscillation in depth during the gradual ascent or bottom phase of dives [42, 43]. We used the ‘zoo’ R package [44] to create a custom function to identify and count peaks of wiggles in the dive profile. This same function was used to measure the amplitude of each wiggle. The function is archived in a GitHub repository provided in the Supplementary Information. Briefly, the function applies a loess smoother across a predefined moving window and identifies the peaks of each ‘wiggle’ using the maximum deviation across consecutive windows. Maximum amplitudes of the wiggles were then calculated using the 95th quantile of the residuals of the dive data fitted with a second loess function (Fig. S2). Different window sizes (w) were used for each individual to account for individual movement patterns and are summarised in Supplementary Table S2.

### Behavioural analysis

For each individual dataset, we recorded the number of dives, total time from the beginning of the first dive to the end of the last dive, total and percentage of time at depth and at the surface, and mean dive duration, post-dive surface interval time, gradual ascent time, maximum dive depth and number of wiggles.

To assess the consistency of U-shaped dives, we identified bouts of consecutive U-shaped dives (≥ 2 dives without interruption by other dive shapes) for each individual. For each bout, we calculated the standard deviation (SD) of maximum depth and total dive duration across all dives within the bout. These metrics were summarised by species to describe intra-bout variability in dive behaviour under the assumption that low variability in dive metrics may reflect stereotyped behaviour such as resting, as has been demonstrated in marine turtles [e.g., 45].

To examine whether inter-dive horizontal distance differs between dive types, a Wilcoxon rank-sum test (Mann–Whitney U test) was used to compare the distances between consecutive surfacing events of U-shaped and S-shaped dives.

We used linear mixed-effects models using the ‘lme4’ package [46] to identify any significant correlations between dive parameters. We also fitted a generalised additive mixed-effects model (GAMM) to explore the relationship between the mean depth of the gradual ascent phase and the maximum depth of the dive using the ‘gamm’ package [47]. Snake ID was included as a random effect to control for repeated measures from the same individual, and species was included as a random effect to control for species-level variation.

To investigate the influence of the diel cycle and tides on the gradual ascent phase, we used a GAMM to examine the relationship between time spent in the gradual ascent phase and hour of the day, and a linear mixed effects model to assess the effect of tide height (measured in 15-minute intervals). Only two individuals were included in these analyses (*H. stokesii* ‘Cruise’ and ‘Dora’) as the *H. major* tracks were too short and the other *H. stokesii* (‘Tony’) did not perform enough S-shaped dives for meaningful conclusions to be drawn. Snake ID was included as a random effect to control for pseudoreplication.

## Results

**Fig. 3:**
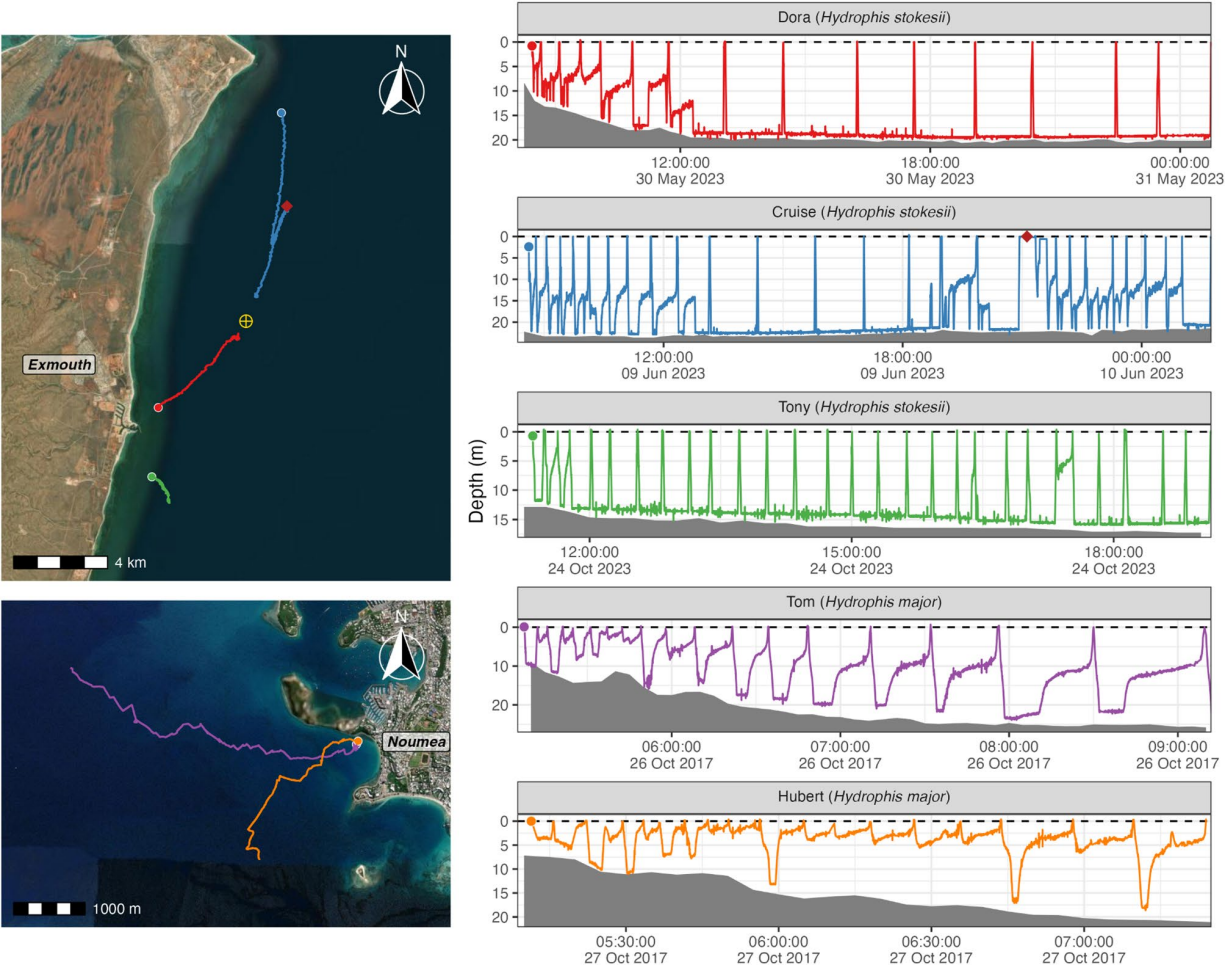
Maps of tracks in (**a**) Exmouth Gulf and (**b**) Baie des Citrons, overlaid on Esri World Imagery basemaps [34], and (**c**) time-depth profiles for each individual included in analyses (*H. stokesii* individuals ‘Dora’, ‘Cruise’, and ‘Tony’ and *H. major* individuals ‘Tom’ and ‘Hubert’). Filled circles mark the start of each track. The yellow circle with a cross indicates the location of King Artificial Reef, and the filled red diamond marks where and when *H. stokesii* ‘Cruise’ was recaptured and re-released. Note that x-axis scales in (**c**) differ between individuals

Eight adult sea snakes were tagged for fine-scale tracking: five *H. stokesii* in Exmouth Gulf and three *H. major* in Baie des Citrons. One *H. stokesii* individual did not acclimate to the external transmitter (repeatedly attempting to dislodge the tag following release) and was therefore promptly recaptured, the transmitter removed, and the animal released in good condition, in accordance with our animal ethics protocols. A second *H. stokesii* individual was excluded from analysis due to its dive profile being a strong outlier, characterised by long surface intervals and short, irregular dives. Although these behaviours may have reflected natural variation, we could not exclude the possibility of influence from the tag or poor weather conditions during the track. One *H. major* individual was also excluded due to poor data quality, including weak signal strength, missing detections, and erratic depth readings, due to difficulties in maintaining consistent tracking. The five tracks included in the analysis (two male and one female *H. stokesii*, and two male *H. major*) ranged from 2.2 to 16.3 h in duration (mean: 9.1 h) after filtering and trimming (Fig. 3).

One snake (*H. stokesii* ‘Cruise’) was recaptured several hours into tracking to re-secure its transmitter which had come loose. The snake resumed diving after release, but the surface interval immediately prior to capture and the first 20 min post-release were excluded from analysis to remove any abnormal behaviour associated with the tagging adjustment (Fig. 3).

### General diving behaviour

A total of 68 dives from three *H. stokesii* individuals and 38 dives from two *H. major* individuals included in the dive analyses. *H. stokesii* reached depths of up to 23 m (the maximum depth of Exmouth Gulf) with an average dive depth of 18.1 ± 4.0 m. The deepest dive by *H. major* was 24 m, with an average dive depth 10.1 ± 6.8 m. The *H. stokesii* individuals dived over seafloors averaging 17.4 ± 3.8 m deep (range 3.5–21.6 m; total tracking time: 2367.7 min). *H. major* individuals dived over seafloors averaging 17.2 ± 5.5 m deep (range 5.2–23.8 m; total tracking time: 374.5 min). The longest dive recorded for *H. stokesii* was 119.8 min (individual ‘Dora’), with a mean duration of 34.2 ± 26.5 min. In comparison, *H. major* dives were shorter, with a maximum duration of 38.9 min (individual ‘Tom’) and a mean dive duration 9.6 ± 8.4 min. Post-dive surface intervals were short for both species: 39.2 ± 30.0 s for *H. stokesii* and 17.1 ± 12.0 s for *H. major*. Overall, individuals spent 98.1% (*H. stokesii*) and 97.1% (*H. major*) of their time at depth. Summary dive metrics for each individual are provided in Table 1, with interspecific comparisons shown in Fig. S3.

**Table 1:** Summary of dive behaviour for each tracked individual. Means are reported with standard deviation

Dive metrics	Dora	Cruise	Tony	Tom	Hubert
Species	*H. stokesii*	*H. stokesii*	*H. stokesii*	*H. major*	*H. major*
Sex	Female	Male	Male	Male	Male
Location	Exmouth Gulf	Exmouth Gulf	Exmouth Gulf	Baie des Citrons	Baie des Citrons
Transmitter attachment	External	External	External	Internal	Internal
Transmitter accuracy	0.5	0.5	0.5	1.7	1.7
Transmitter resolution	0.15	0.15	0.15	0.2	0.2
Total length (cm)	146.5	87.5	123	107	110
Snout-vent length (cm)	129	73	106	-	-
Weight (g)	2665	415	1573	840	930
Total dives	15	25	28	18	20
Surfacing rate (per hr)	0.92	1.63	3.60	4.46	9.05
Total S-shaped dives	6	19	2	12	12
Total U-shaped dives	9	6	26	6	8
Total time (min)	978.7	922.8	466.2	241.9	132.6
Total time at depth (min)	963.9	905.1	454.5	237.3	126.5
Proportion of time at depth	0.98	0.98	0.97	0.98	0.95
Total time at surface (min)	14.8	17.7	11.7	4.6	6.2
Proportion of time at surface	0.02	0.02	0.03	0.02	0.05
Mean dive duration (min)	64.3 ± 31.5	36.2 ± 20.5	16.2 ± 4.2	13.2 ± 10.6	6.3 ± 3.6
Mean surface interval (min)	1.0 ± 0.4	0.7 ± 0.6	0.4 ± 0.3	0.3 ± 0.1	0.3 ± 0.2
Mean gradual ascent (min)	8.5 ± 12.2	12.2 ± 9.8	0.5 ± 1.8	6.0 ± 7.0	2.8 ± 3.1
Mean bottom time (min)	51.3 ± 39.9	18.2 ± 26.8	13.6 ± 5.5	3.8 ± 2.9	1.7 ± 1.4
Mean max dive depth (m)	17.2 ± 4.0	22.3 ± 0.6	14.7 ± 1.7	13.4 ± 7.3	7.1 ± 4.7
Proportion of S-shaped dives with wiggles	1.0	0.95	0.5	-	-
Mean number of wiggles per S-dive	11	7.6	1.5	-	-
Mean max wiggle amplitude (m)	0.56 ± 0.19	0.75 ± 0.28	0.37 ± 0	-	-

### Dive shapes

**Fig. 4:**
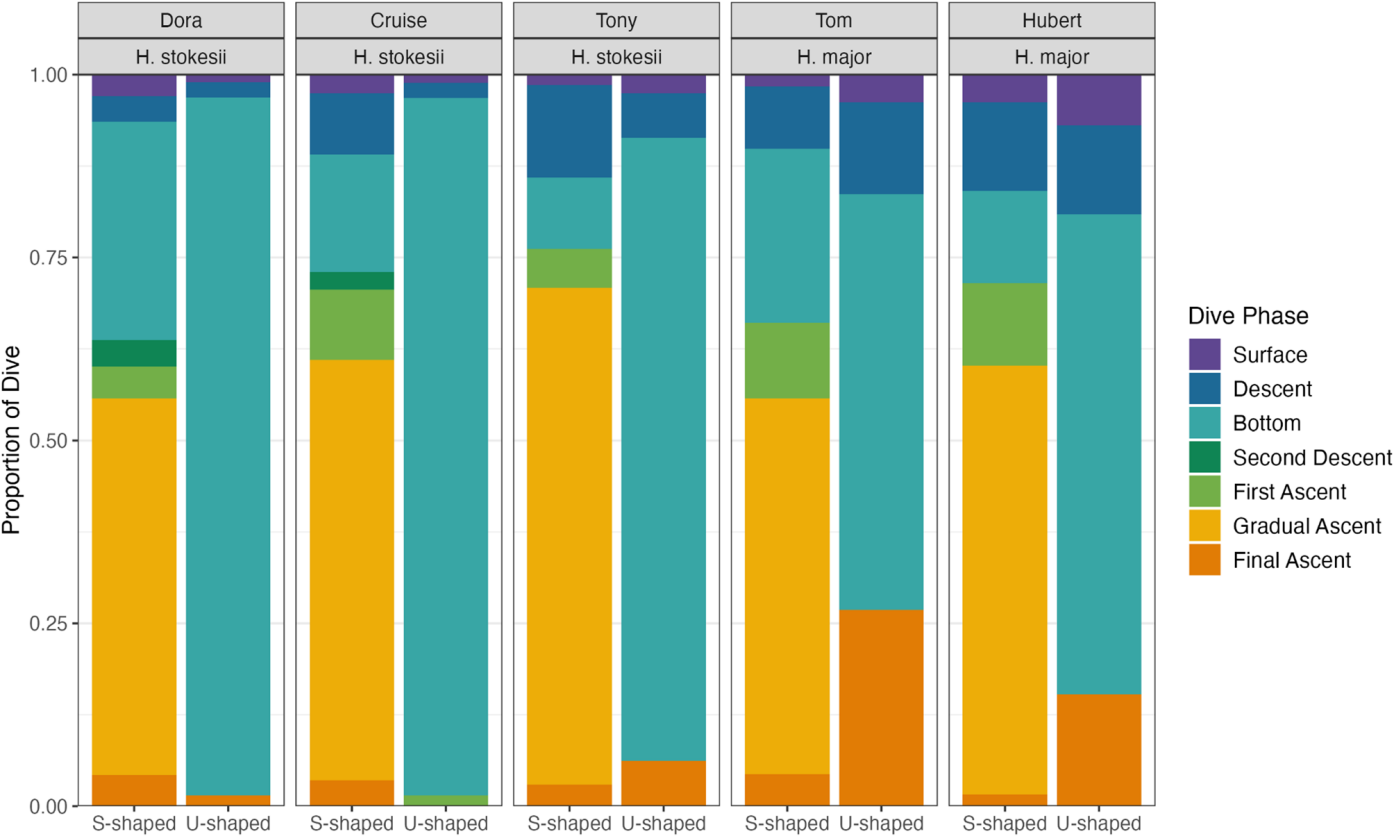
Proportion of dive phases that made up S- and U-shaped dives for sea snake individuals

All snakes conducted both U-shaped and S-shaped dives. U-shaped dives were more frequent in *H. stokesii* (60.3%), though dive shape frequencies varied considerably between individuals (Table 1). In contrast, the two *H. major* displayed a higher proportion of S-shaped dives (63.2%) than U-shaped dives (36.8%). S-shaped dives generally included a prolonged gradual ascent phase, whereas U-shaped dives were dominated by bottom time (Fig. 4). In *H. stokesii*, U-shaped dives were longer in duration (37.6 ± 31.3 min) than S-shaped dives (28.8 ± 16.1 min). In *H. major*, however, S-shaped dives were longer (12.4 ± 9.3 min) than U-shaped dives (4.9 ± 1.9 min), suggesting they may serve different or additional functions in this species.

With the exception of three outliers (identified as values > 1.5 × IQR), U-shaped dives conducted by *H. stokesii* had a standard deviation (SD) of depth during the bottom phase (a proxy for activity level) of less than 0.5 m. This falls within the estimated depth sensor accuracy of the transmitters used for this species (mean = 0.12 m; range = 0.05–0.23 m), suggesting that most U-shaped dives were associated with stationary or resting behaviour. However, the three U-shaped dives (7% of *H. stokesii* U-dives) identified as outliers had bottom-phase SDs of 2.08, 0.96, and 0.70 m, and may reflect foraging activity. In contrast, U-shaped dives by *H. major* showed a higher mean bottom-phase SD (mean = 0.43 m; range = 0.14–1.00 m), though interpretation is limited by the lower accuracy of the transmitters used for this species (± 1.7 m), which exceeds the observed variability in depth.

We identified seven bouts of consecutive U-shaped dives (≥ 2 dives) across both species. *H. stokesii* performed four U-shaped bouts, with an average of 9.5 dives per bout. These bouts were relatively consistent in depth (mean depth SD = 0.28 m, median = 0.15 m), but more variable in duration (mean duration SD = 9.9 min, median = 9.0 min). In contrast, *H. major* exhibited three U-shaped bouts, with an average of 3.3 dives per bout. These were more variable in depth (mean depth SD = 2.61 m, median = 3.28 m), but more consistent in duration (mean duration SD = 1.1 min, median = 1.0 min).

**Fig. 5:**
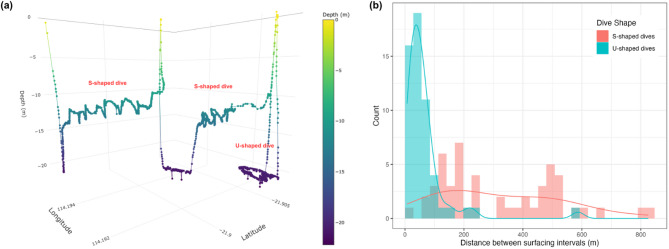
(**a**) Three-dimensional plot of two S-shaped and one U-shaped dive completed by *H. stokesii* ‘Cruise’, with points coloured by depth. (**b**) Histogram of distances between surfacing intervals (inter-dive distances) coloured by dive shape

A Wilcoxon rank-sum test revealed a significant difference in inter-dive distances between U-shaped and S-shaped dives (W = 2627, *p* < 0.001; Fig. 5b). U-shaped dives were associated with much shorter distances between surfacing events (median = 48.1 m, IQR = 45.7 m) compared to S-shaped dives (median = 240.0 m, IQR = 340.0 m), indicating distinct spatial patterns linked to dive shape (Fig. 5a).

Although most dives could be broadly classified as U- or S-shaped dives, high variability was observed between dives. For instance, several S-shaped dives consisted of a prolonged period on the seafloor before or after the gradual ascent phase. Some dive profiles included a brief ascent of a few metres followed by a brief descent back to the same depth within the gradual ascent or bottom phase.

Most S-shaped dives performed by *H. stokesii* involved intricate wiggling during the gradual ascent phase (Fig. 2). Three or more wiggles were identified in 92% of *H. stokesii* S-shaped dives and there was a mean of 7.9 ± 4.8 wiggles per dive. Mean maximum wiggle amplitude for all individuals was 0.69 m (individual means are reported in Table 1). The *H. major* individuals tracked in Baie des Citrons were equipped with an older transmitter model with lower depth sensor resolution and accuracy (± 1.7 m; Table 1), which prevented reliable detection of wiggling behaviour, as fine-scale depth changes could not be distinguished from sensor noise.

### Correlations between dive parameters

**Fig. 6:**
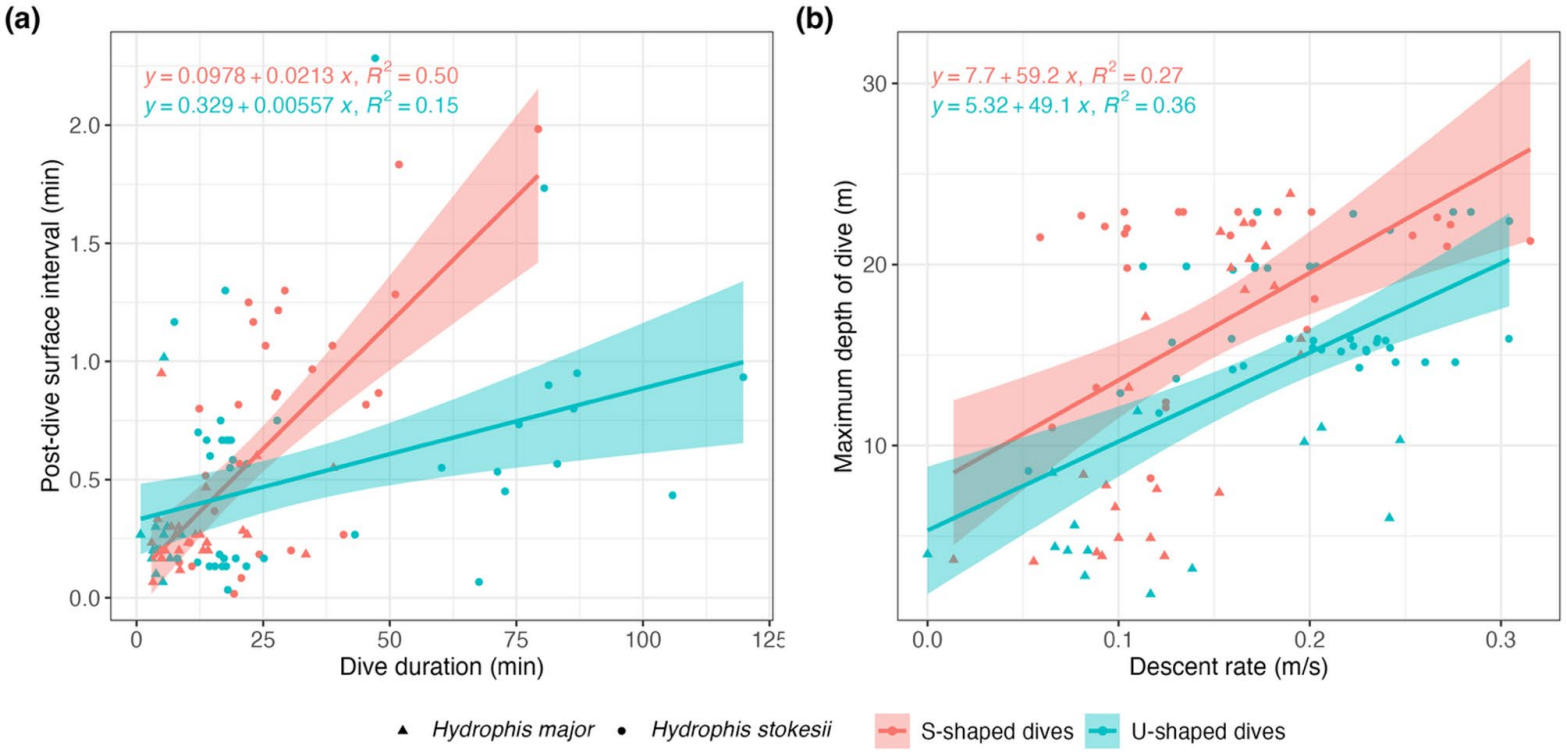
Dive parameters of *H. major* (triangles) and *H. stokesii* (circles) for U-shaped dives (red lines) and S-shaped dives (blue lines). (**a**) Post-dive surface interval as a function of dive duration. (**b**) Maximum depth of dive as a function of descent rate

**Fig. 7:**
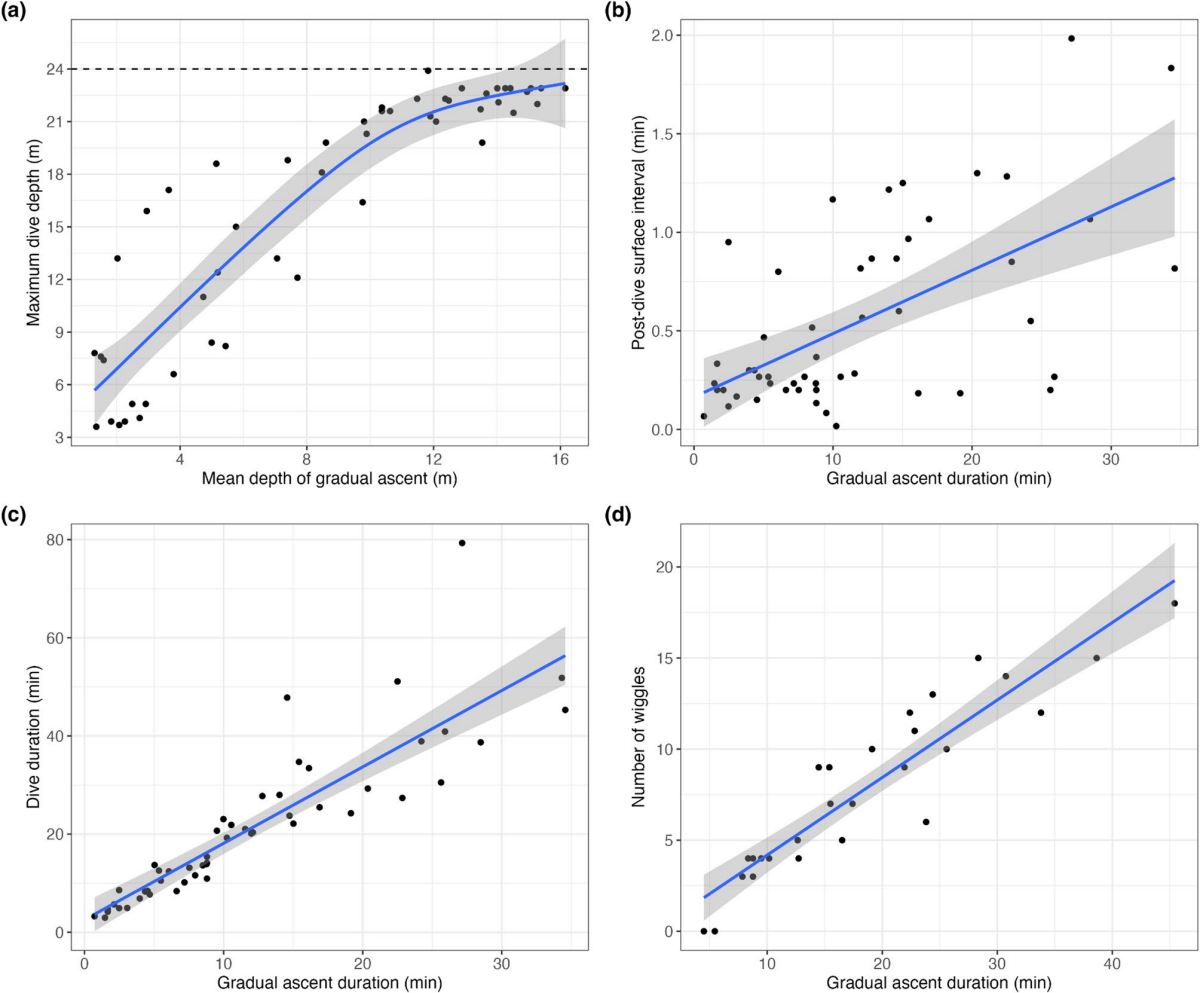
Gradual ascent dive parameter correlations for *H. stokesii* and *H. major*. (**a**) Mean depth of the gradual ascent phase as a function of the maximum depth of the dive. Dashed line indicates the greatest maximum depth of the environment recorded during all tracks. (**b**) Post-dive surface interval as a function of gradual ascent duration. (**c**) Dive duration as a function of gradual ascent duration. (**d**) Number of wiggles as a function of gradual ascent duration

Dive durations were significantly and positively correlated with post-dive intervals (β = 0.02, 95% CI [0.01, 0.03], *t*_(100)_ = 4.98, *p* < 0.001), though this relationship was weaker for U-shaped dives compared to S-shaped dives, as indicated by a significant interaction effect (β = -0.02, 95% CI [-0.02, -7.65e-03], *t*_(100)_ = -4.02, *p* < 0.001; Fig. 6a). While U-shaped dives tended to have longer post-dive intervals than S-shaped dives, this main effect was not statistically significant (β = 13.65, 95% CI [-0.59, 27.89], *t*_(100)_ = 1.90, *p* = 0.060). A Generalised Additive Mixed-effects Model (GAMM) indicated that the mean depth of the gradual ascent phase was predicted by and positively correlated with the maximum depth of the dive (R^2^ = 0.83, deviance explained = 90.96%, *p* < 2e-16; Fig. 7a). Positive correlations were also found between dive duration and maximum depth of a dive (conditional R^2^ = 0.83; *t*_(100)_ = 0.12; *p* = 0.91; Fig. S4), dive duration and gradual ascent duration (conditional R^2^ = 0.70; *t*_(102)_ = 2.67; *p* < 0.009; Fig. 7c), post-dive surface interval and gradual ascent time (conditional R^2^ = 0.36; *t*_(102)_ = 3.70; *p* < 0.001; Fig. 7b), and descent rate and maximum depth of a dive (conditional R^2^ = 0.44; *t*_(100)_ = 2.88; *p* = 0.005; Fig. 6b). Finally, the number of wiggles during a gradual ascent was positively correlated with its duration (conditional R^2^ = 0.66; *t*_(21)_ = -0.75; *p* = 0.459; Fig. 7d), and the number of wiggles was positively correlated with maximum wiggle amplitude (conditional R^2^ = 0.33; *t*_(21)_ = 1.93; *p* = 0.067; Fig. S5).

### Gradual ascent phase in S-shaped dives

In *H. stokesii*, the gradual ascent phase began 5.44 ± 2.45 m and finished 10.07 ± 2.36 m above the seafloor. The average vertical distance travelled during this phase was 4.42 ± 1.53 m over 16.5 ± 8.6 min. The gradual ascent phase made up an average of 59.1% of each S-dive. The rate of ascent was the slowest of all vertical transit rates (0.006 ± 0.006 m/s).

In comparison, *H. majo*r began the gradual ascent phase 9.34 ± 1.99 m above the seafloor, ascending to a distance of 13.19 ± 2.91 m before initiating the final ascent. They travelled an average vertical distance of 3.34 ± 1.92 m over this 6.8 ± 5.6 min during this phase. The gradual ascent phase contributed to an average of 52.2% of total dive time for S-shaped dives. The rate of ascent was again the slowest vertical transit rate (0.010 ± 0.005 m/s).

Linear models indicated that tidal height was negatively correlated with gradual ascent duration, indicating *H. stokesii* may spend less time in this phase during high tides, however the relationship was not significant (conditional R^2^ = 0.19; *t*_(130)_ = 0.96; *p* = 0.339; Fig. S6). There was no significant relationship between gradual ascent duration and time of day (R^2^ = 0.01, *p* = 0.27; Fig. S7).

## Discussion

This study used high-resolution spatial movement data to reveal novel insights into the fine-scale diving patterns of two benthic-foraging sea snake species. It represents only the second application of active acoustic telemetry to characterise diving behaviour in free-swimming sea snakes, and the first for benthic-foraging species [15]. We successfully recorded 45.7 h of fine-scale data, capturing 106 dives from five individuals across two species and locations. This small sample size reflects the inherent difficulty of locating, capturing, tagging, and tracking sea snakes over extended periods. Of the eight individuals tagged, three were excluded due to failure to acclimate to external tags or poor data quality resulting from challenging tracking conditions. One snake required re-tagging due to a loose attachment, and another was released approximately 8.5 km from its capture location to avoid poor weather conditions. These interferences may have influenced movement patterns and should be considered when interpreting the results. Additionally, although sea snakes were briefly held in aquaria before tagging and release to accommodate weather and crew availability, both this holding period and the tagging method (internal vs. external) may have influenced behaviour. These potential effects remain uncertain and warrant further investigation in future studies. Despite these limitations, the data analysed in this study provide novel and valuable insights into the underwater behaviours of sea snakes, which we discuss in detail below.

### General diving behaviour

In the current study, both species of *Hydrophis* dived continuously, spending nearly all their time submerged (95–98%) and very little time at the surface (Table 1). This high submergence rate is comparable to previous observations by Rubinoff et al. [15] in the uniquely pelagic yellow-bellied sea snake, *Hydrophis platurus*, with proportions of the fifteen tracked individuals ranging from 51 to 99.9%. Similarly, observations of continuous diving behaviour have also been observed in benthic-foraging sea kraits, which have been attributed to maximising foraging time near the seafloor [39]. Unlike amphibious sea kraits, which rest in shelter sites on land, sea snakes remain at sea indefinitely and therefore must spend some of their time resting either on the surface or seafloor.

Average dive durations by tracked *H. stokesii* (ca. 33 min, up to 120 min) and *H. major* (ca. 10 min, up to 40 min) align broadly with estimates from other *Hydrophis* species [12, 48], though species-specific and environmental factors likely shape these patterns. For example, free-ranging *H. platurus* has been recorded diving for up to 213 min [15], while *L. saintgironsi* displays much shorter submergence times of just 9.0 ± 5.7 min (0.2–37.8 min) [39]. Differences between *H. major* and *H. stokesii* are likely driven by the contrasting habitats in which they were tracked; however, longer tracking durations and larger sample sizes are needed to reliably compare interspecific dive capacities. There was also considerable individual variation in most dive metrics (Table 1), suggesting that inter-individual differences, such as body condition or recent activity, could influence dive patterns.

Post-dive surface intervals were short for both species, averaging ca. 31 s in *H. stokesii* and 17 s in *H. major*, suggesting rapid gas exchange at the surface, possibly supported by cutaneous respiration while submerged [49]. These intervals were much shorter than those recorded for *H. platurus* (S- and V-shaped dives were reported separately as ca. 3.2 min and ca. 8.4 min, respectively), and *L. saintgironsi* (all dives reported as ca. 2.4 min), likely due to differences in foraging mode and physiology [19, 39]. Both *H. stokesii* and *H. major* are commonly observed floating passively on the surface at night (and *H. stokesii* during the day, particularly when injured or consuming large prey) (Coppersmith pers. obs.), but the function of this behaviour remains unknown.

### Dive shapes

Both species performed only U- and S-shaped dives in the current study. In penguins, seabirds, pinnipeds and turtles, U-shaped dives are often interpreted as foraging behaviour based on their frequency, tendency to occur in bouts at uniform depths, and their diel variation in maximum depth [50]. In marine turtles, however, U-shaped dives can also reflect resting on the seafloor [10, 51–53], and changes in depth or activity during the bottom phase are commonly used to distinguish between foraging and resting behaviours [8].

Our three-dimensional data suggest that most U-shaped dives performed by sea snakes were more likely associated with resting. Individuals remained at consistent depths and in the same location during these dives, as indicated by short distances between consecutive surfacing events. Movement along the seafloor was also limited, and some of this apparent movement may reflect drift of the vessel around a stationary snake rather than active horizontal displacement. Furthermore, consecutive U-shaped dives were relatively consistent in depth and duration, particularly in *H. stokesii*, supporting the interpretation of resting behaviour [45]. In this species, U-shaped dives were also generally longer in duration than S-shaped dives, further supporting this interpretation. Sea snakes are commonly observed resting on the seafloor and often anchor themselves between pieces of coral or hide in crevices [9, 54]. In contrast, the S- and V-shaped dives observed in *H. platurus* suggest that this species does not rest on the seafloor, likely due to its deep, open ocean habitats [19].

S-shaped dives, the other dive type observed in this study, appear to reflect energy-efficient movement through the water column. These dives featured a long gradual ascent phase, accounting for an average of 56.3% of total dive duration, consistent with gliding at neutral buoyancy with minimal energy expenditure [19, 22, 55]. Similar dive profiles have been described in *H. platurus* [15, 56] and several sea turtle species [e.g., 8, 20–22]. Unlike most air-breathing marine mammals, sea snakes and marine turtles inhale rather than exhale before diving [3]. They therefore must overcome positive buoyancy during descent and their depth of neutral buoyancy changes with the volume of air inhaled [57, 58]. Without neutral buoyancy, it is unlikely that an animal could remain at the same depth in the water column without high energetic costs [19]. In our study, the narrow vertical depth range of the gradual ascent phase (average ca. 4.28 and 3.24 m for *H. stokesii* and *H. major*, respectively) supports the interpretation of passive gliding during this portion of the dive.

Interestingly, sea snakes initiated S-shaped dives by first descending to the sea floor, though the function of this behaviour is not entirely clear. While U-shaped dives likely function as resting or foraging dives, the initial bottom contact in S-shaped dives may serve other roles, such as orientation, habitat assessment, or testing buoyancy. The maximum dive depths recorded were relatively shallow (< 25 m), likely constrained by local bathymetry. Although the diving limits of *H. stokesii* and *H. major* are unknown, *Hydrophis* sea snakes are regularly observed in deeper waters (> 80 m), with two individuals unidentifiable to species-level having been recorded at maximum depths of 239–245 m [59]. Other *Hydrophis* species including *H. coggeri*, *H. kingii*, and *H. peronii* have been recorded at maximum depths of 60 m and *H. ocellatus* as deep as 112 m [60]. At those depths, these snakes likely experience negative buoyancy, requiring considerable energetic expenditure to ascend to depths of neutral and positive buoyancy.

Dive shape frequencies varied considerably between individuals (Table 1). Identifying the drivers of these differences (such as habitat or time since last feeding) may be possible with additional fine-scale tracking data. Approximately two thirds of dives were S-shaped for both *H. major* individuals, though fewer dives were recorded for this species (*n* = 38), limiting generalisations. The bottom phase of S-shaped dives varied considerably in duration (22 s to 45 min), potentially indicating that sea snakes perform multiple tasks within a single dive [e.g., resting and foraging; 8].

### Dive parameter correlations

Given that the gradual ascent phase is likely where sea snakes are neutrally buoyant, we would expect its depth to remain constant if the same volume of air was inhaled before each dive. However, as *H. stokesii* and *H. major* performed deeper dives, the mean depth of their gradual ascent phase increased (Fig. 7a), suggesting they regulate inspired air volume based on intended dive depth or environmental depth. This ability to “plan” a dive has also been recorded in seabirds [61–63], sea turtles [38, 57, 58, 64, 65], and *H. platurus* [56, 66]. Seabirds adjust air volume to meet oxygen demands, while sea turtles and *H. platurus* regulate buoyancy via depth-specific inhalation [38, 56–58, 64, 65]. Our data suggest that benthic-foraging sea snakes use a similar strategy to minimise oxygen consumption and the energetic costs of swimming against positive or negative buoyancy. This is supported by the positive relationship between the maximum depth of a dive and its duration (Fig. S4), likely reflecting greater air volumes, and thus more oxygen, during deeper dives. By the same logic, before completing U-shaped dives, sea snakes likely intake a certain volume of air that allows them to rest easily on the seafloor with slightly negative buoyancy, potentially relying more on cutaneous oxygen uptake to sustain longer submergence times. Although the weaker positive correlations observed between dive duration and post-dive surface intervals for U-shaped compared to S-shaped dives is consistent with this hypothesis, lung volume experiments are needed to test it. A positive correlation was also observed between descent rate and the maximum depth of a dive (Fig. 6b), suggesting snakes must swim faster to overcome greater positive buoyancy resulting from the greater inflation of their lungs.

Dive duration was positively correlated with post-dive surface interval (Fig. 6a), possibly reflecting a need for multiple respiratory cycles to offload accumulated CO_2_ and replenish oxygen stores following longer dives. This relationship was significant for S-shaped dives, when snakes are presumably more active, but weaker and non-significant for U-shaped dives, which were mostly associated with resting behaviour and likely involve lower metabolic rates. A similar pattern was found in *L. saintgironsi* for dives < 10 min [39], but not in *H. platurus*, likely due to their high levels of cutaneous gas exchange [19, 67]. However, green sea turtles show longer surface durations following resting dives than foraging or migratory dives, because they expend nearly all stored oxygen to maximise dive duration for energetic efficiency [10]. The positive correlation between the gradual ascent duration and post-dive surface interval (Fig. 7b) may indicate that this phase is aerobically demanding. However, given the strong correlation between dive duration and gradual ascent duration (Fig. 7c), the longer surface intervals may simply reflect the longer recovery needed after longer dives, especially since snakes are neutrally buoyant during this phase. It is still unclear whether sea snakes use longer post-dive intervals to repay ‘oxygen debts’ accumulated through anaerobic respiration. Sea snakes have rarely been observed in the wild to take more than one breath at the surface before resuming diving [68] and have been found to increase surfacing rates, rather than breathing bouts, during periods of elevated metabolic demand [48]. This is consistent with their limited oxygen storage in muscle or blood compared to the lungs, indicating a single exhale and inhale may be enough to replenish oxygen stores in most situations [15].

### Potential functions of the gradual ascent phase in S-dives

Given the high likelihood that sea snakes are neutrally buoyant in the gradual ascent phase and can regulate inhaled air volume to control the depth of neutral buoyancy, the question of why they choose to be neutrally buoyant several metres above the seafloor remains. Cook & Brischoux [19] suggested that S-shaped dives in *H. platurus* may be used to avoid predators, unfavourable surface conditions, or to locate oceanic slicks (in which they forage) while travelling efficiently. However, as *H. stokesii* and *H. major* are benthic foragers, this phase may serve different functions. For example, the depth of the gradual ascent could reflect thermal gradients, favourable temperatures, or subsurface currents. Given the large horizontal distances and direct travel during this phase, we suggest it facilitates efficient transit between habitats, potentially through passive gliding at neutral buoyancy. Neutral buoyancy has been shown to minimise the energetic cost of horizontal swimming in other marine vertebrates, such as seals [69], and may confer similar benefits in sea snakes. For instance, *H. stokesii* ‘Dora’ completed S-shaped dives until reaching within 500 m of an artificial reef, where she switched to U-shaped dives (Fig. 3). Travelling ~ 6 m above the seafloor may allow sea snakes to move uninterrupted across patchy habitats and over reef structures that would otherwise obstruct their path if swimming closer to the seafloor. It may also enable passive drifting on subsurface currents while avoiding surface threats.

Alternatively, sea snakes may maintain neutral buoyancy in the water column to reduce predation risk. Predators of sea snakes include sharks [70, 71], teleosts [12] and seabirds [72, 73]. Notably, sea snakes are key prey for tiger sharks in Shark Bay, Western Australia and New Caledonia [74–76]. Although swimming in the water column may provide protection from aerial predators or large fishes, it could also increase exposure to tiger sharks, which oscillate vertically in search of prey [77, 78]. Furthermore, if predator avoidance were the main driver, we would expect sea snakes to swim closer to the seafloor, where reef structures offer refuge. Further study on sea snake predator interactions is needed to fully evaluate this hypothesis.

Another possibility is that the gradual ascent phase supports habitat or prey searching. Although observational data sea snake foraging is limited, some species (e.g., *Emydocephalus annulatus*, *Aipysurus laevis*, *A. duboisii*) locate their prey by scent, tongue-flicking into crevices [14, 79], while others (*H. melanocephalus*, *H. ornatus*) use both olfaction and vision [80]. In the present study, ‘interrupted’ S-shaped dives, where individuals briefly return to the seafloor during the gradual ascent (Fig. 2), may reflect foraging or searching behaviour. However, whether sea snakes can distinguish habitat from the depth of gradual ascents (ca. 7.8 m above the seafloor in *H. stokesii*, ca. 11.3 m in *H. major*) remains uncertain. Recent work suggests visual abilities in some species are more advanced than previously thought [81, 82], and photoreceptor densities vary among species [83]. For example, *H. major* has higher densities on the ventral portion of their retinas compared to *A. laevis*, potentially enabling them to scan for predators above [56]. However, if visual or chemosensory acuity were driving gradual ascent depth, we might expect sea snakes to swim closer to the seafloor. Additionally, *Hydrophis* sea snakes lack rod-type photoreceptors for dim-light vision, potentially limiting visual searching to daylight hours [83, 84]. Despite this, we found no diel pattern in S-shaped dives (Fig. S6 & S7), although the short track durations (< 24 h) may have limited our ability to detect existing patterns.

### Wiggling

Many of the gradual ascents performed by *H. stokesii* featured repeated vertical oscillations of up to 1.3 m (Video S1). Such undulations, known as ‘wiggles’, have been recorded in penguins, seabirds, pinnipeds and turtles [e.g., 50, 85, 86, 87], where they are often linked to foraging, particularly in birds where wiggles correlate with beak movements [88, 89]. However, the wiggles observed in *H. stokesii* differ in several respects. They were only observed during the gradual ascent phase in S-shaped dives–rather than at the bottom of U-shaped dives–and were generally uniform in amplitude and duration, occurring consistently throughout the gradual ascent rather than sporadically. Although *H. major* may perform similar movements, the lower resolution of the sensors used during their tracks prevented detection of such fine-scale behaviours.

While *H. stokesii* occasionally consumes midwater fishes such as porcupine fish, its diet is primarily benthic. We therefore conclude that wiggles are unlikely to represent prey pursuits. Instead, they may function as a searching technique to scan the water column for sensory cues, as has been suggested in southern elephant seals [90]. Although oscillating such a small vertical distance (ca. 0.69 m; Fig. S5) is unlikely to enhance visual searching, the repetitive motion is consistent with olfactory search patterns in other animals [91]. For instance, sharks and salmon use low-amplitude zig-zag movements (side-to-side in sharks, up-and-down in salmon) to track odour patterns in turbulent flows and water layers [92].

Alternatively, wiggling could aid in buoyancy control, particularly as it occurred only during the phase where sea snakes are likely neutrally buoyant. Vertical oscillations may allow snakes to test or fine-tune their depth of neutral buoyancy or calibrate their position in the water column for efficient or rapid horizontal travel. Direct observations of free-ranging sea snakes swimming during the gradual ascent phase are limited (e.g. Video S2), and further studies incorporating acceleration data and improved understanding of their swimming mechanics are needed to clarify the function of this behaviour.

## Conclusions

This study provides the first high-resolution insights into the fine-scale diving behaviours of benthic-foraging sea snakes, revealing distinct dive shapes linked to different functions. These findings contribute to a more nuanced understanding of the behavioural strategies sea snakes use to manage the energetic and physiological demands of life underwater. Although some interspecific differences were observed, limited sample size and site variation precluded formal comparisons. Future work should track multiple species across a broader range of habitats over longer durations to identify species-specific differences in diving behaviour. Nevertheless, our results highlight the value of fine-scale, three-dimensional data for interpreting the function of dive components and provide a baseline for future comparative studies. Understanding such dive strategies enhances our broader knowledge of the ecological adaptations of secondarily aquatic reptiles.

## Supplementary Information

Below is the link to the electronic supplementary material.

Supplementary Material 1

Supplementary Material 2

Supplementary Material 3

## Data Availability

All datasets generated and analysed, and custom R scripts used in the current study are available in the following GitHub repository https://github.com/vinayudyawer/seasnake-wigglecounter.
