# Supplementary material for: Wiggle and glide: fine-scale telemetry reveals unique diving strategies in benthic-foraging sea snakes
Source: Mov Ecol. 2025 Aug 28;13:62. doi: 10.1186/s40462-025-00592-z (PMC12395837; doi:10.1186/s40462-025-00592-z)
Supplement: Supplementary file 1 — Supplementary Material 1 [file 40462_2025_592_MOESM1_ESM.docx]

**Supplementary Information**


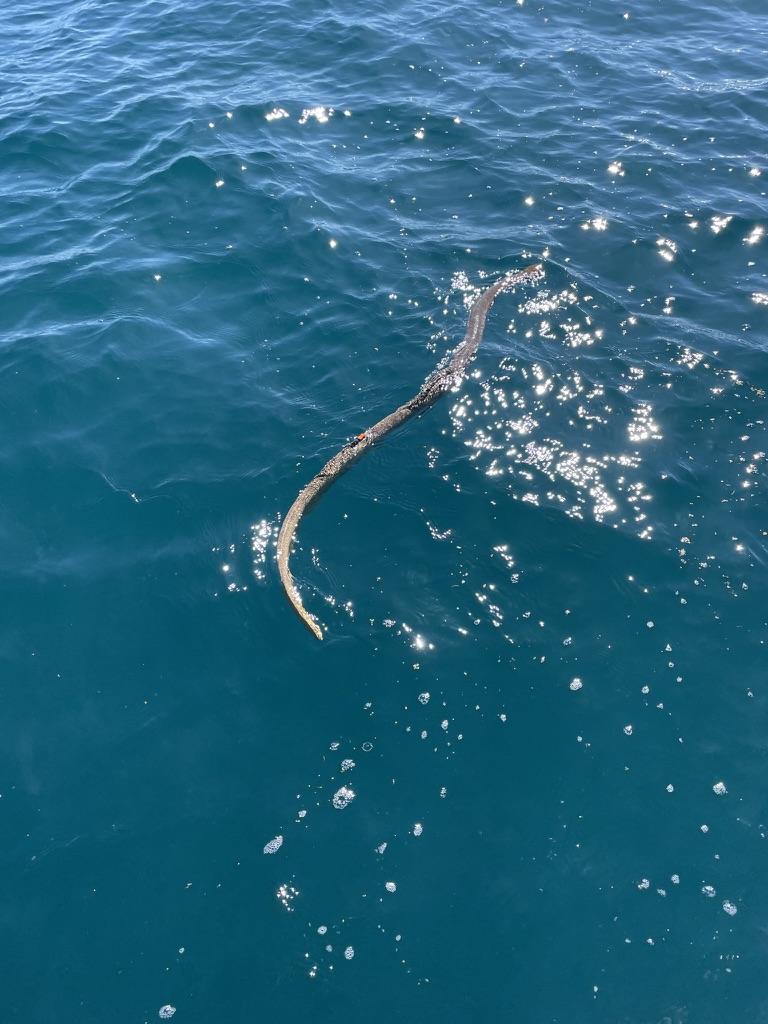

Figure S1: Photo of acoustic transmitter externally attached to *H. stokesii* ‘Dora’.

Table S1: Capture and release metadata (including ID, species, data captured, date tracked, capture location and distance from capture location) for each tracked sea snake.

| **Snake ID** | **Dora** | **Cruise** | **Tony** | **Tom** | **Hubert** |
| --- | --- | --- | --- | --- | --- |
| Species | *H. stokesii* | *H. stokesii* | *H. stokesii* | *H. major* | *H. major* |
| Date captured | 28/5/2023 | 8/6/2023 | 23/10/2023 | 23/10/2017 | 23/10/2017 |
| Date tracked | 30/5/2023 | 9/6/2023 | 24/10/2023 | 26/10/2017 | 27/10/2017 |
| Capture location | -21.958387, 114.14308 | -21.756317, 114.215567 | -21.976067, 114.13535 | -22.29543, 166.4367 | -22.29802, 166.4378 |
| Release location | -21.957483, 114.146967 | -21.831733, 114.19905 | -21.974317, 114.141633 | -22.296126, 166.436021 | -22.296126, 166.436021 |
| Distance from capture location (km) | 0.41 | 8.56 | 0.68 | 0.11 | 0.27 |
| PIT | 445442 | 445419 | 927371 | – | – |
| Sex | F | M | M | M | M |
| Weight (g) | 2665 | 415 | 1555 | 840 | 930 |
| SVL (cm) | 146.5 | 73 | 106 | – | – |
| TL (cm | 129 | 87.5 | 123 | 107 | 110 |

​​Table S2: Filtering thresholds applied to each individual, including the minimum signal strength (dB) and maximum and minimum depth (m) for detections to be included, as well as the total percentage of detections filtered out using these thresholds. The window size (W) used to identify and count wiggles during the gradual ascent phase is shown in the bottom row.

| **ID** | **Dora** | **Cruise** | **Tony** | **Tom** | **Hubert** |
| --- | --- | --- | --- | --- | --- |
| Min signal strength (dB) | 65 | 65 | 50 | 45 | 43 |
| Max depth (m) | 20 | 23 | 16 | 24 | 22 |
| Min depth (m) | -0.5 | -0.5 | -0.5 | -0.5 | -0.5 |
| Window size (W) | 12 | 16 | 18 | - | - |


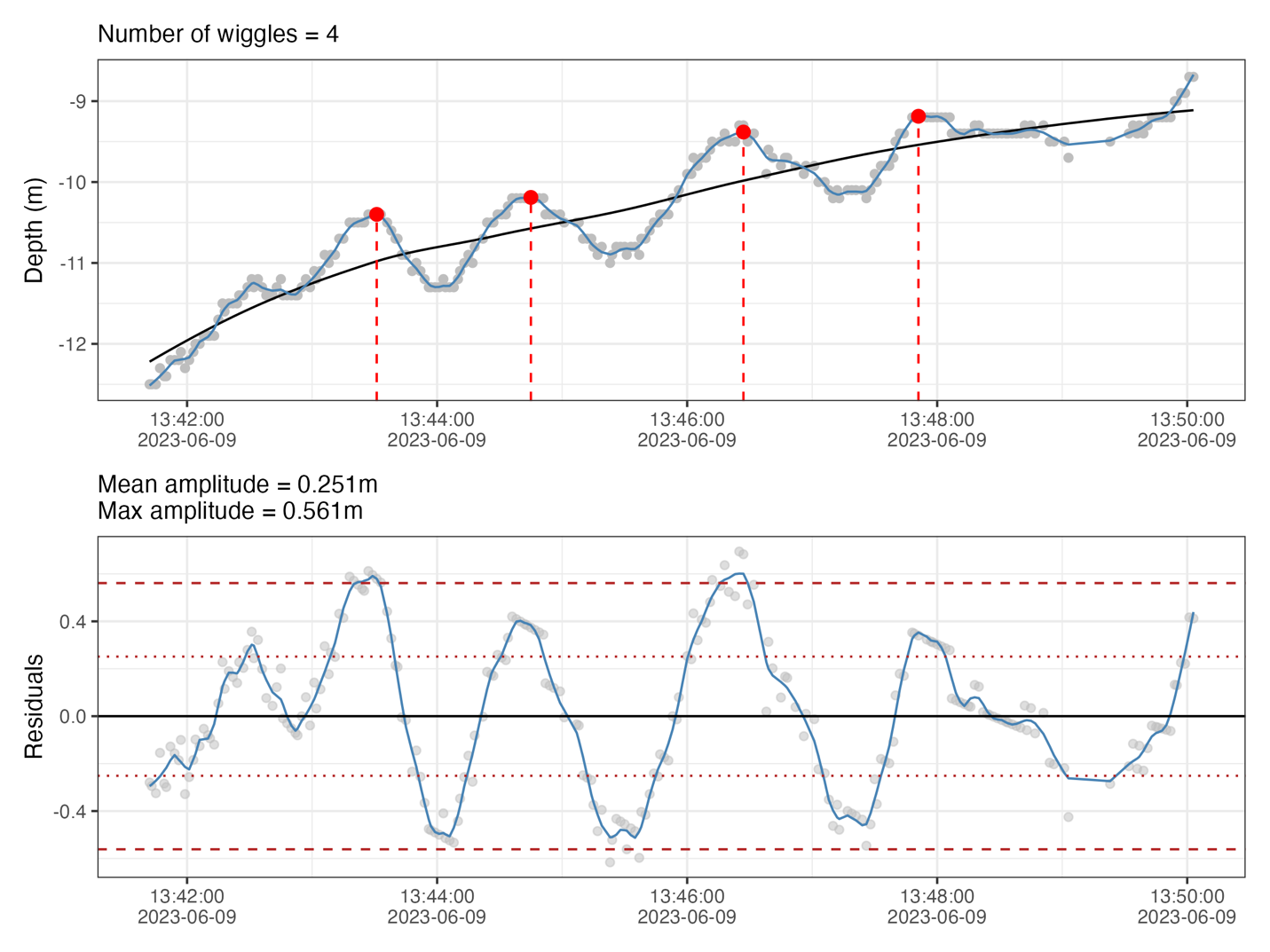


Figure S2: A visualisation of the ‘wiggle counter’ custom function (which can be found at the GitHub repository here: https://github.com/vinayudyawer/seasnake-wigglecounter) which was designed to identify peaks in the gradual ascent phase of S-shaped dives.


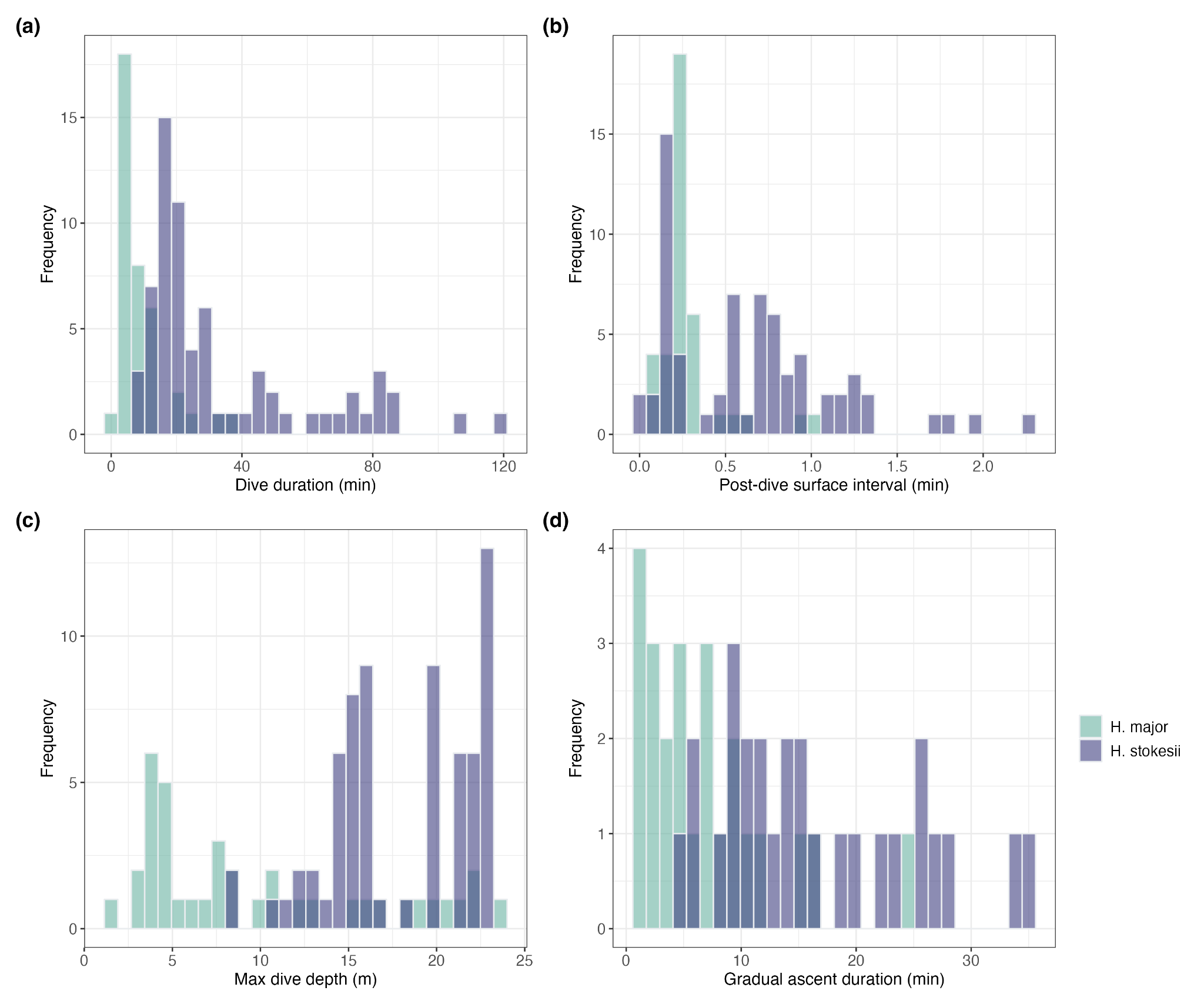


Figure S3: Histograms depicting dive duration, post-dive interval, gradual ascent duration (for S-shaped dives only), gradual ascent rate, descent rate, and maximum depth for *H. stokesii* and *H. major*.


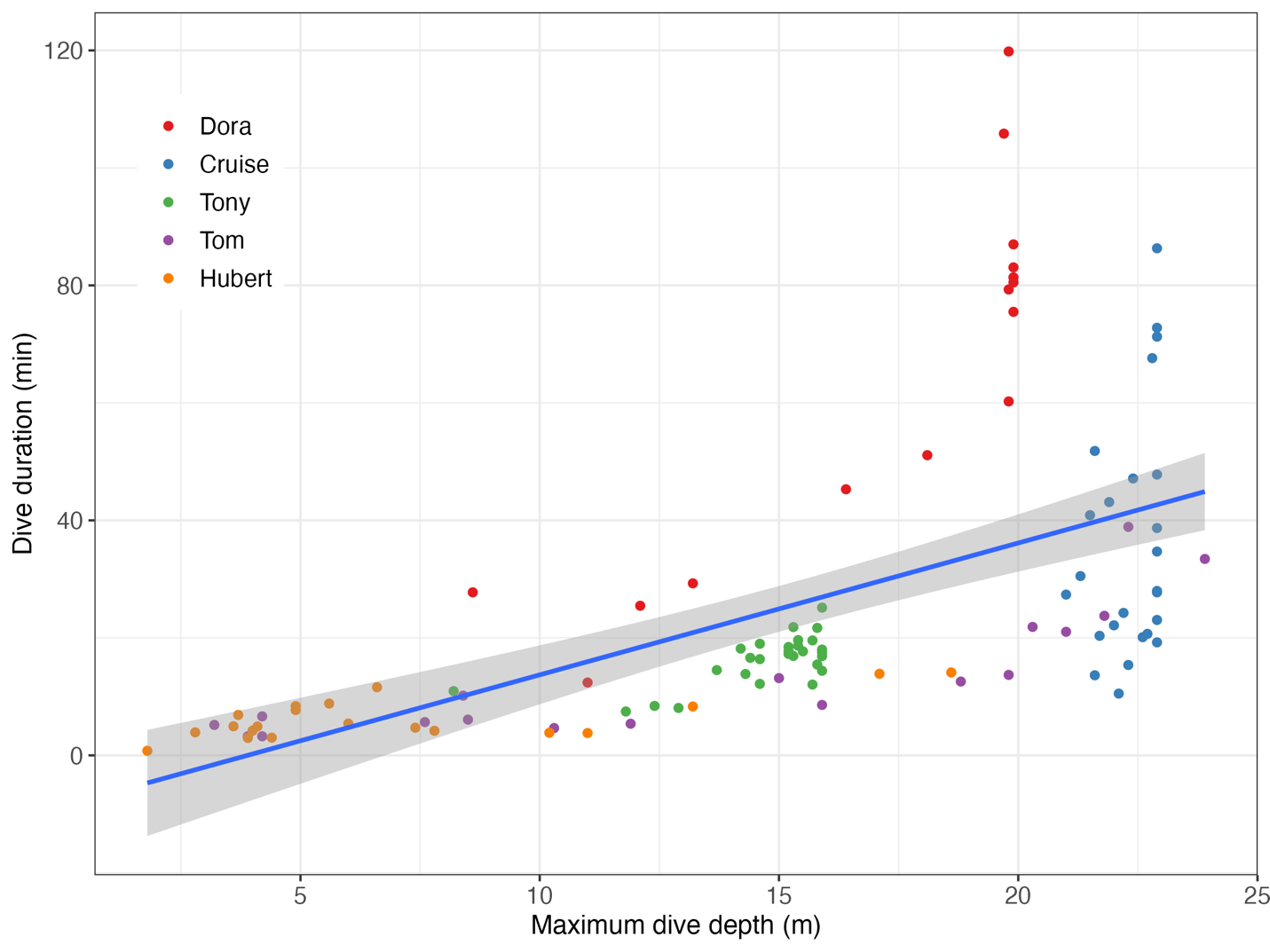
Figure S4: Scatterplot showing a positive linear relationship between dive duration (minutes) and maximum dive depth (metres). Each point represents a single dive, coloured by individual.


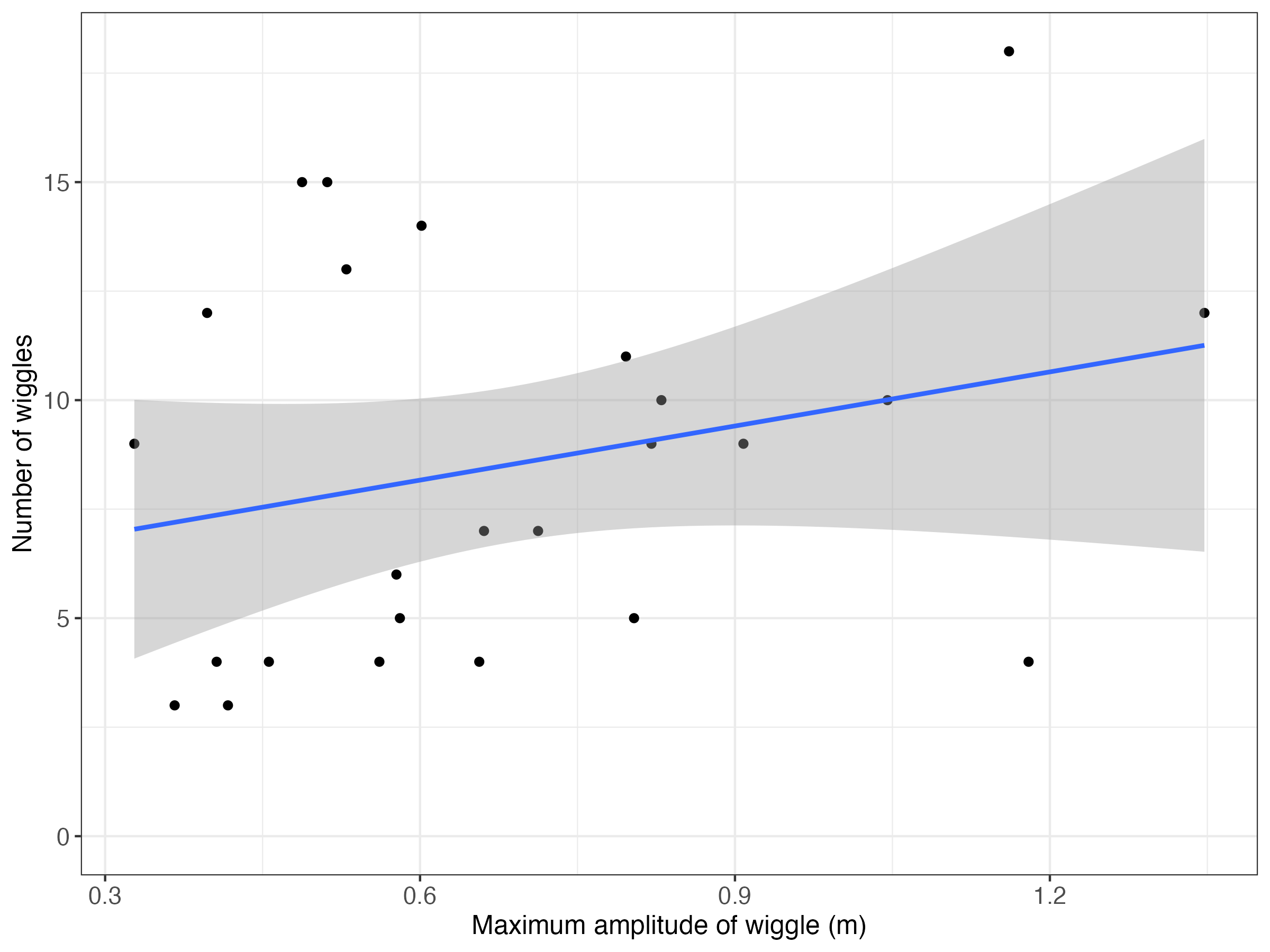
Figure S5: Number of wiggles recorded during each gradual ascent (where wiggles were observed) performed by all *H. stokesii* individuals, plotted as a function of the maximum wiggle amplitude (calculated using the custom ‘wiggle counter’ function; see Figure S2) during the same ascent.


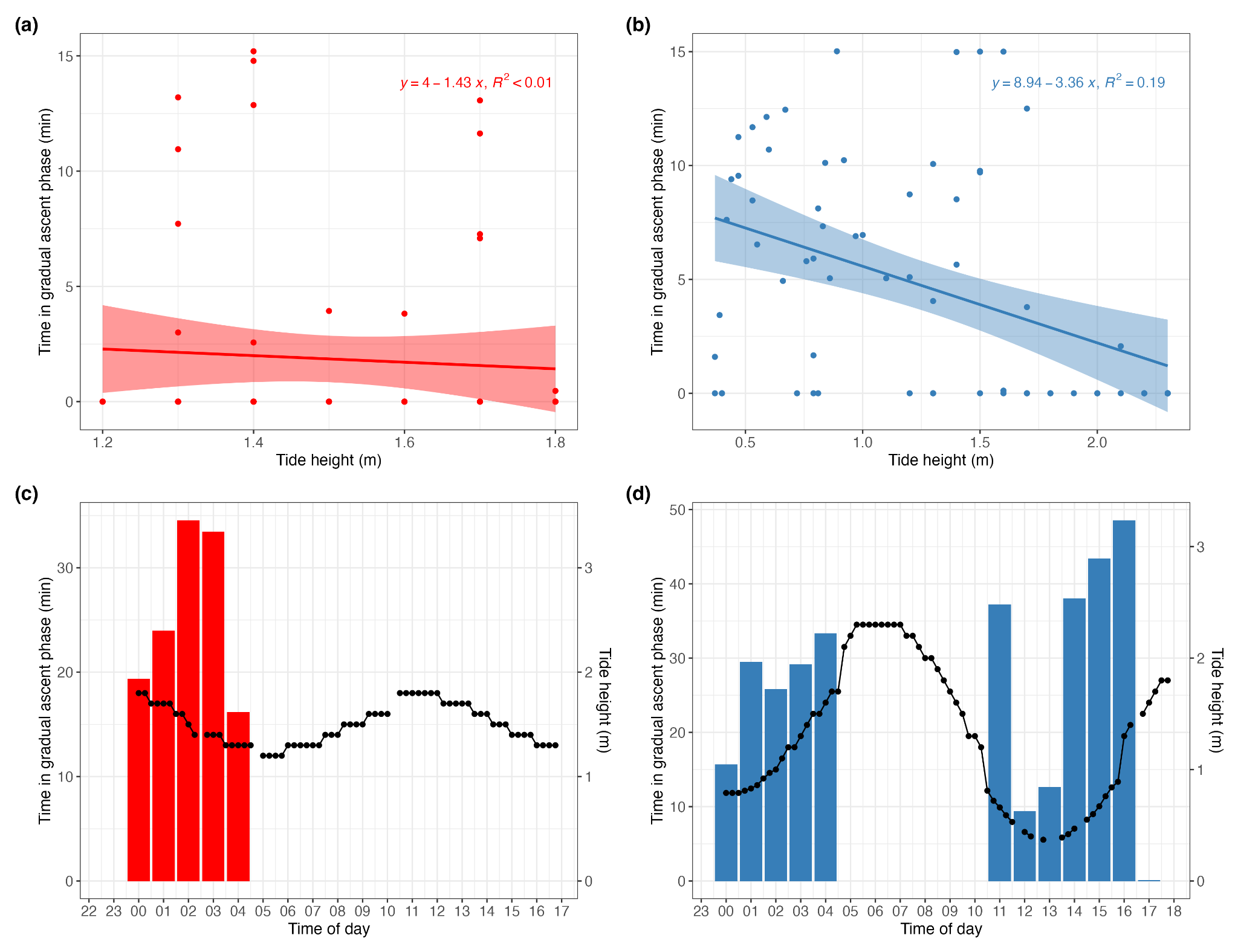


Figure S6: Plots depicting relationships between the time *H. stokesii* ‘Dora’ (red) and *H.stokesii* ‘Cruise’ (blue) spent in the gradual ascent phase each hour of the day. Plots (a) and (b) are tidal height as a function of time in gradual ascent phase and (b) and (c) show time in gradual ascent phase for each hour tracked (bars) and the tidal height (black dots).


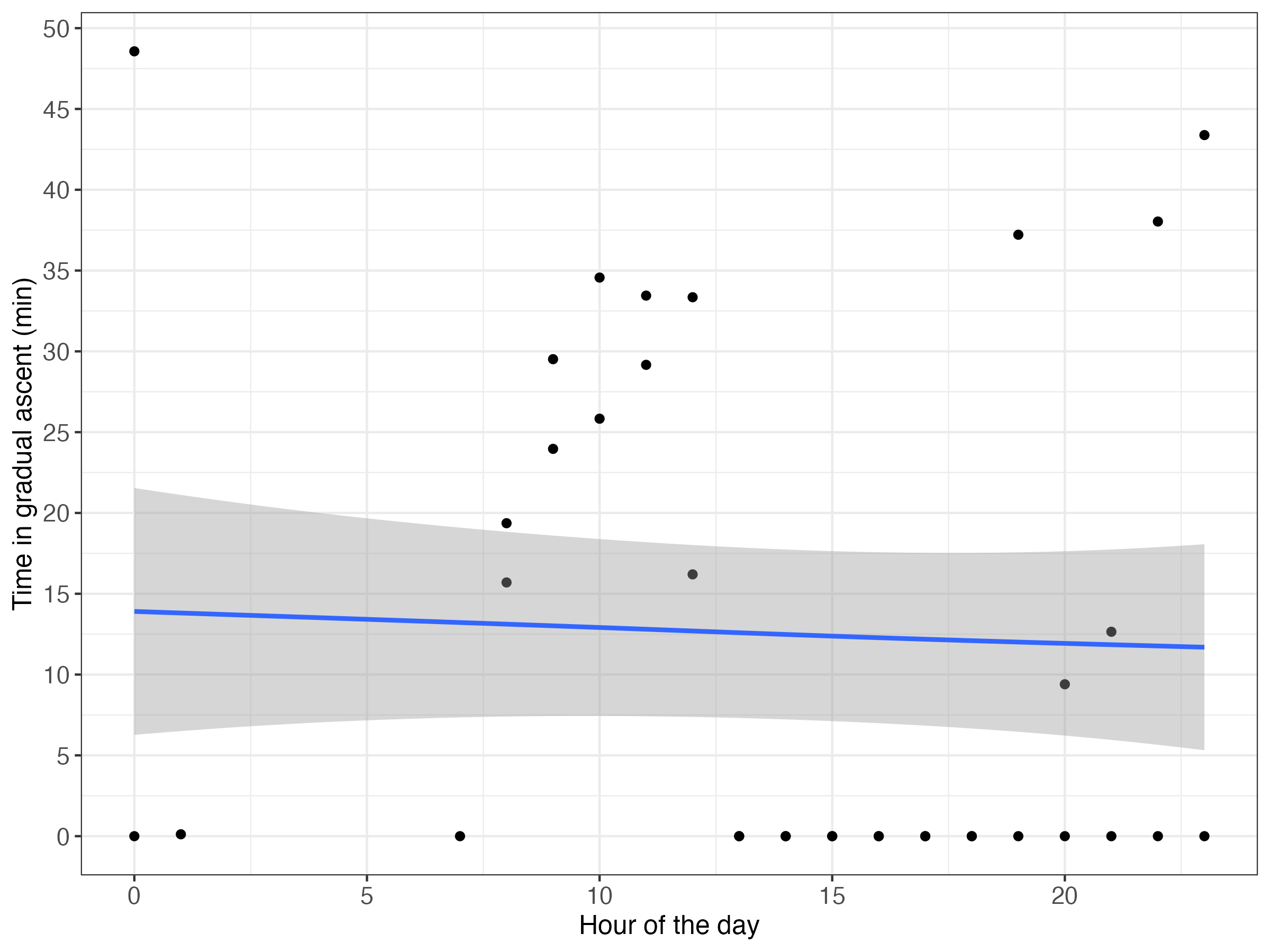
Figure S7: Total time all *H. stokesii* and *H. major* individuals spent in the gradual ascent phase during each hour of the day (0–24).

Video S1: Animation of the dive track of *Hydrophis stokesii* ‘Cruise’, illustrating characteristic wiggling during the gradual ascent phase of S-shaped dives, and minimal seafloor movement during U-shaped dives. Some apparent horizontal displacement may reflect vessel drift rather than active movement by the snake.

Video S2: BRUVS video showing two free-ranging *Hydrophis* sea snakes swimming in the middle of the water column.
